# Inhibition of IRE1/JNK pathway in HK‐2 cells subjected to hypoxia‐reoxygenation attenuates mesangial cells‐derived extracellular matrix production

**DOI:** 10.1111/jcmm.15964

**Published:** 2020-10-11

**Authors:** Yan Liang, Lulu Liang, Zhenjie Liu, Yingzi Wang, Xiubing Dong, Lingyun Qu, Rong Gou, Yulin Wang, Qian Wang, Zhangsuo Liu, Lin Tang

**Affiliations:** ^1^ Department of Nephrology The First Affiliated Hospital of Zhengzhou University Zhengzhou China; ^2^ Research Institute of Nephrology Zhengzhou University Zhengzhou China; ^3^ Key Laboratory of Precision Diagnosis and Treatment for Chronic Kidney Disease in Henan Province Zhengzhou China; ^4^ Core Unit of National Clinical Medical Research Center of Kidney Disease Zhengzhou China; ^5^ Department of Geriatrics The First Affiliated Hospital of Zhengzhou University Zhengzhou China

**Keywords:** AKI‐CKD transition, ECM, hypoxia‐reoxygenation, inflammation, IRE1, JNK

## Abstract

Endoplasmic reticulum (ER) stress and inflammatory responses play active roles in the transition of acute kidney injury (AKI) to chronic kidney disease (CKD). Inositol‐requiring enzyme 1 (IRE1) activates c‐Jun NH_2_‐terminal kinase (JNK) in ER stress. Tubular epithelial cells (TEC) are the main injury target and source of AKI inflammatory mediators. TEC injury may lead to glomerulosclerosis, however, the underlying mechanism remains unclear. Here, hypoxia/reoxygenation (H/R) HK‐2 cells were used as an AKI model. To determine the partial effects of TEC injury on the glomerulus, HK‐2 cells after H/R were co‐cultured with human renal mesangial cells (HRMC). H/R up‐regulated ER stress, IRE1/JNK pathway, IL‐6 and MCP‐1 in HK‐2 cells. Stimulation of HRMC with IL‐6 enhanced their proliferation and the expression of glomerulosclerosis‐associated fibronectin and collagen IV via signal transducer and activator of transcription 3 (STAT3) activation. Similar responses were observed in HRMC co‐cultured with HK‐2 cells after H/R. IRE1/JNK inhibition reversed these injury responses in HRMC. IRE1/JNK stable knock‐down in HK‐2 cells and shRNA‐mediated STAT3 depletion in HRMC confirmed their role in inflammation/glomerulosclerosis. These findings suggest that IRE1/JNK pathway mediates inflammation in TEC, affecting mesangial cells. The inhibition of this pathway could be a feasible approach to prevent AKI‐CKD transition.

## 1 INTRODUCTION

Acute kidney injury (AKI) is a global public health concern characterized by the rapid loss of renal function, associated with high morbidity, mortality and healthcare costs.^1^, ^2^ AKI has been recognized as a major risk factor for chronic kidney disease (CKD) and end‐stage kidney disease (ESRD).^3^ According to a study developed in the USA, Canada, Western Europe and Australia, approximately 1.5 million people suffering from AKI survive each year. Among them, 15%‐20% progress to advanced CKD within 24 months, resulting in approximately 300 000 cases of advanced CKD each year.^4^ According to a nationwide survey in China, approximately 3 million adult AKI patients are cured in hospitals across the country every year, of note, around 50% develop CKD, resulting in 1.5 million CKD‐cases per year.^4^ Moreover, another study estimated that 20% of patients experiencing an episode of AKI would develop CKD within 3 years.^5^ Even the milder form of AKI can have adverse outcomes and may lead to renal fibrosis, the final pathway to CKD and ESRD.^3^ The mechanisms of AKI‐CKD transition are extremely complex and include maladaptive repair in renal tubules, endoplasmic reticulum (ER) stress, inflammation, mitochondrial damage, cell cycle arrest, autophagy and senescence.^6^, ^7^, ^8^, ^9^, ^10^, ^11^ The ER stress and inflammatory response, as well as maladaptive repair appear to be critical mediators of chronic histological changes.^12^, ^13^

Endoplasmic reticulum stress has been implicated in the pathogenesis of both AKI and CKD.^14^, ^15^, ^16^, ^17^ In response to cellular stress, unfolded or misfolded proteins accumulate in the ER, triggering the unfolded protein response, which involves three ER stress sensors, double stranded RNA activated protein kinase like ER kinase (PERK), activating transcription factor‐6 (ATF6) and inositol‐requiring enzyme 1 (IRE1).^1^ The activation of IRE1 stimulates the c‐Jun NH_2_‐terminal kinase (JNK) pathway, which regulates cell survival and secretion of pro‐inflammatory cytokines to promote the characteristic inflammatory milieu.^18^

It is reported that specific TEC injury could lead to glomerulosclerosis. The accumulation of extracellular matrix (ECM) in the glomerulus is an important feature of glomerulosclerosis.^3^ The main ECM cell‐secretors in glomerulus are mesangial cells. Recent studies using db/db mice have shown that the activation of the signal transducer and activator of transcription 3 (STAT3) pathway in mesangial cells by IL‐6 leads to the secretion of fibronectin (FN), which, in turn, promotes renal fibrosis and sclerosis.^19^, ^20^, ^21^ Therefore, the IL‐6/STAT3 pathway may mediate inflammatory damage in mesangial cells and glomerular ECM, which may promote AKI‐CKD transition.^11^ However, the mechanism behind remains largely unclear. To study the effect of TEC on mesangial cells in AKI‐CKD transition, we stably knocked down IRE1α or JNK1 in human renal proximal tubular epithelial cells (HK‐2 cells) and exposed them to hypoxia/reoxygenation (H/R) injury. Afterwards, we silenced STAT3 in human renal mesangial cells (HRMC), stimulated them with IL‐6 and co‐cultured HRMC with HK‐2 cells. Our findings demonstrate that IRE1α or JNK1 silencing in H/R HK‐2 cells attenuate chronic injuries in HRMC. Mechanistically, inhibiting the IRE1/JNK pathway weakened inflammation, STAT3 signalling, cell proliferation and ECM production. Thus, the modulation of the IRE1/JNK pathway may prevent glomerulosclerosis and represent a potential therapeutic strategy to impede AKI‐CKD transition.

## 2 METHODS

### 2.1 Cell culture and treatment

#### 2.1.1 HK‐2 cells

HK‐2 cells were purchased from ATCC: The Global Bioresource Center. These cells were cultured with Dulbecco's Modified Eagle Medium (DMEM)/F12 medium (Gibco, Thermo Fisher Scientific) supplemented with 10% foetal bovine serum (FBS, Gibco), 100 U/mL penicillin (Geneview) and 100 µg/mL streptomycin (Geneview) in a humidified incubator with 5% CO_2_ at 37°C. The subculture of HK‐2 cells was performed using 0.25% Trypsin‐EDTA (Gibco). To induce a hypoxia‐reoxygenation injury model, HK‐2 cells with serum‐free DMEM/F12 were exposed to 4 hours of hypoxia (5% CO_2_, 1% O_2_, 94% N_2_ at 37°C), followed by 6, 12 or 24 hours of reoxygenation (5% CO_2_, 95% air at 37°C) in normal culture medium. Controls were incubated in a humidified incubator with 5% CO_2_ at 37°C. IRE1α or JNK1 knock‐down HK‐2 cell lines (stable depletion) were generated by Obio Technology Corp. Ltd. The two stable cell lines were incubated with 0.4 µg/mL puromycin (Solarbio) in DMEM/F12 medium. Of note, the knock‐down efficiency of both genes was greater than 70%. In some experiments, these cells were subjected to H/R treatment.

#### 2.1.2 HRMC

Human renal mesangial cells were purchased from ScienCell™. These cells were cultured as reported previously.^22^ Briefly, cells were plated on poly‐l‐lysine‐coated culture vessels with mesangial cell medium (MsCM) (ScienCell™) supplemented with 2% FBS (ScienCell™), 100 U/mL penicillin, 100 μg/mL streptomycin (ScienCell™) and 1% mesangial cell growth factors (ScienCell™). Following starvation in serum‐free MsCM for 24 hours, cells were washed and stimulated with 0.1 ng/mL human IL‐6 (200‐06; Peprotech) for 12, 24 or 36 hours.^23^ Control cultures were incubated with MsCM only.

#### 2.1.3 STAT3 knock‐down in HRMC

The STAT3 shRNA adenovirus tagged with a GFP fluorescent protein was constructed by the Han Heng Biotechnology Company. STAT3 shRNA adenovirus transfection was carried out to knock‐down STAT3 gene expression in HRMC. In brief, the STAT3 shRNA adenovirus (multiplicity of infection, MOI = 100) was added to 50% confluent HRMC cells, and cells were incubated for 6 hours. Following transfection, the culture media was removed and HRMC were cultured with fresh medium for 24 hours. Thereafter, cells were stimulated with 0.1 ng/mL human IL‐6 for another 24 hours. Control HRMC were transfected with a control adenovirus without STAT3 shRNA using the same method. Cells were subsequently harvested and prepared for further analysis. The top strand of STAT3 shRNA was: AATTCGTGGACAATATCATTGACCTTGTGAATTCAAGAGATTCACAAGGTCAATGATATTGTCCATTTTTTG. The bottom strand of the STAT3 shRNA was: GATCCAAAAAATGGACAATATCATTGACCTTGTGAATCTCTTGAATTCACAAGGTCAATGATATTGTCCACG.

#### 2.1.4 Co‐culture system

Trans‐well co‐culturing was performed using six‐well polystyrene plates (Corning, Inc) and inserts with permeable membranes having a pore size of 0.4 µm. HRMC were seeded into the lower chamber, and HK‐2 cells in the upper chamber (insert). Stable HK‐2 cell lines with control lentivirus or depletion, were cultured in hypoxic conditions for 4 hours, and then were moved into the wells containing HRMC and both cell types were co‐cultured in reoxygenation for 24 hours. HRMC were digested for testing. The experimental groups were as follows: (a) HRMC co‐cultured with HK‐2 cells infected with control lentivirus, (b) HRMC co‐cultured with HK‐2 cells infected with IRE1α or JNK1 knock‐down lentivirus, (c) HRMC co‐cultured with H/R HK‐2 cells infected with control lentivirus, (d) HRMC co‐cultured with H/R HK‐2 cells infected with IRE1α or JNK1 knock‐down lentivirus.

### 2.2 Western blotting

Cells were lysed in radioimmunoprecipitation assay buffer supplemented with protease phosphatase inhibitors. Protein samples were processed for immunoblot analysis as previously reported.^23^ The following primary antibodies were used: β‐actin (AP0060; Bioworld Technology), GRP78 (ab21685; Abcam), PERK (ab65142; Abcam), ATF6 (65880; Cell Signaling Technology), IRE1α (CST3294), pIRE1α(phosphorylation sites:S724) (ab124945; Abcam), JNK (CST9252), pJNK(phosphorylation sites:Thr183/Tyr185) (CST4668), CHOP (ab179823; Abcam), STAT3 (CST12640), pSTAT3(phosphorylation sites:Tyr705) (CST9145), Collagen IV (ab6586; Abcam), FN (ab2413; Abcam). The working concentration of β‐actin was 1:5000, and that of other antibodies was 1:1000. Secondary antibodies were obtained from Dingguo and were used at the final dilution of 1:5000.

### 2.3 Detection of cytokines by enzyme‐linked immunosorbent assay (ELISA)

HK‐2 cell culture supernatants were collected and subsequently centrifuged for 25 minutes at 2500 rpm and 4°C. We used a sandwich ELISA kit for the measurement of IL‐6 (R&D Systems) and MCP‐1 (R&D Systems), following the manufacturer's instructions.

### 2.4 Transmission electron microscopy

Following H/R, HK‐2 cells were trypsinized and centrifuged at 1000 rpm for 10 minutes. Thereafter, cells were soaked with 2% glutaraldehyde for a minimum of 1 hours at 4°C. Cells were then thoroughly washed with 0.1 mol/L phosphate buffer saline (PBS, without disrupting the cell pellet). Following decanting of the supernatant, 1% osmium acid (in PBS) was added and the mixture left standing for 1.5 hours. Following another washing step, cells were dehydrated with increasing concentrations of acetone (30%, 50%, 70%, 90% and 100%) and embedded in 812 resin (Canemco, 034). The cell structure and organelles, including the ER were observed using a JEOL JEM‐1400 Plus Transmission Electron Microscope (JEOL USA, Inc).

### 2.5 RNA extraction and quantitative reverse transcription‐polymerase chain reaction (qRT‐PCR)

Total RNA was extracted from cells using a commercial RNA Extraction Kit (Qiagen) according to the manufacturer's protocol. cDNA was synthesized (from RNA templates) using the Revert Aid First Strand cDNA Synthesis Kit (Thermo Fisher Scientific). Subsequently, qRT‐PCR was performed in triplicate using the Maxima SYBR Green qPCR Master Mix (2X) with a separate ROX vial (Thermo Fisher Scientific) and the SLAN‐96P Real‐Time PCR System. mRNA levels were normalized to those of the housekeeping gene actin‐beta (ACTB). The primers used are listed in Table 1.

**TABLE 1:** Primer sequences used in this study (qRT‐PCR)

GENE	Primer sequence	
CHOP	Forward	5′‐GGTACCTATGTTTCACCTCCTGG‐3′
	Reverse	5′‐CTCCTCAGTCAGCCAAGCCA‐3′
IL‐6	Forward	5′‐CAATGAGGAGACTTGCCTGGTG‐3′
	Reverse	5′‐TGGCATTTGTGGTTGGGTCA‐3′
MCP‐1	Forward	5′‐CTCGCTCAGCCAGATGCAAT‐3′
	Reverse	5′‐CACTTGCTGCTGGTGATTCTTCT‐3′
IRE1α	Forward	5′‐GCAAGAGGACAGGCTCAATCA‐3′
	Reverse	5′‐GATTCCATCTGAACTTCGGCA‐3′
JNK1	Forward	5′‐AGCCAGTCAGGCAAGGGATT‐3′
	Reverse	5′‐ATTGATGTACGGGTGTTGGAGA‐3′
COL4A1	Forward	5′‐GGCACAGGACCTTTGGGAGA‐3′
	Reverse	5′‐CCGGGCTGACATTCCACAA‐3′
FN	Forward	5′‐GCCGAATGTAGGACAAGAAGC‐3′
	Reverse	5′‐GCCGAATGTAGGACAAGAAGC‐3′
STAT3	Forward	5′‐GAGAAGGACATCAGCGGTAAGAC‐3′
	Reverse	5′‐TTGGTCTTCAGGTATGGGGCA‐3′
ACTB	Forward	5′‐CACCCAGCACAATGAAGATCAAGAT‐3′
	Reverse	5′‐CCAGTTTTTAAATCCTGAGTCAAGC‐3′

### 2.6 Immunofluorescence assay

Human renal mesangial cells were cultured onto cover slips in 6‐well plates and then exposed as previously described. At the end of exposure, the cells were fixed in 4% paraformaldehyde for 15 minutes at room temperature. Slides were washed in PBS for three times, the target preparation was defined using a liquid blocker pen. Subsequently, the target area was incubated with 3% BSA at room temperature for 30 minutes, and then with primary antibodies FN (ab2413; Abcam) and Col IV (ab6586; Abcam), overnight at 4°C, in a wet box. After three washing steps with PBS (pH 7.4), cell climbing slides were incubated with secondary antibodies, FITC conjugated goat anti‐rabbit IgG (GB22303; Servicebio) or Cy3 conjugated goat anti‐rabbit IgG (GB21303; Servicebio), at room temperature for 50 minutes. and then cells were stained with 0.2 mg/mL DAPI and analysed using a Zeiss LSM 880 confocal microscope (Carl Zeiss).

### 2.7 HRMC proliferation assessment using the cell counting kit‐8 assay

Human renal mesangial cell were seeded in 96‐well tissue culture plates (Costar) at a density of 1 × 10^5^ cells/mL in 100 µL MsCM. Following starvation in serum‐free MsCM for 24 hours, cells were washed and stimulated with 0.1 ng/mL human IL‐6 for 12, 24 or 36 hours. Controls were incubated with MsCM only. Cell proliferation was assessed using the cell counting kit‐8 (CCK‐8) (Dojindo). Briefly, 10 µL of CCK‐8 was added to each well and incubated at 37°C for 2 hours. Thereafter, the absorbance was measured at 450 nm using a microplate reader (iMark; Bio‐Rad). In the co‐culture system, HRMC were co‐cultured with H/R HK‐2 cells in six‐well plates for 24 hours. Subsequently, the method described above was used to detect HRMC proliferation.

The experiments were performed in triplicate and repeated three times. The % cell proliferation was calculated as follows:

$$\%\;\text{cell}\;\text{proliferation}\;=\;\frac{\text{OD}\;(\text{test})\;‐\;\text{OD}\;(\text{blank})}{\text{OD}\;(\text{control})\;‐\;\text{OD}\;(\text{blank})}\;\times \;100$$

### 2.8 Statistical analyses

Normally distributed data, representative of at least three independent experiments, are expressed as the mean ± standard deviation (SD). For continuous variables, independent‐sample *t* tests and one‐way ANOVA tests were performed for comparisons between two, or more than two groups, respectively. A *P*‐value of <.05 was considered significant. Analyses were performed using the SPSS software version 17.0 (SPSS Inc).

## 3 RESULTS

### 3.1 H/R‐induce ER stress and inflammatory cytokines in HK‐2 cells

**FIGURE 1:**
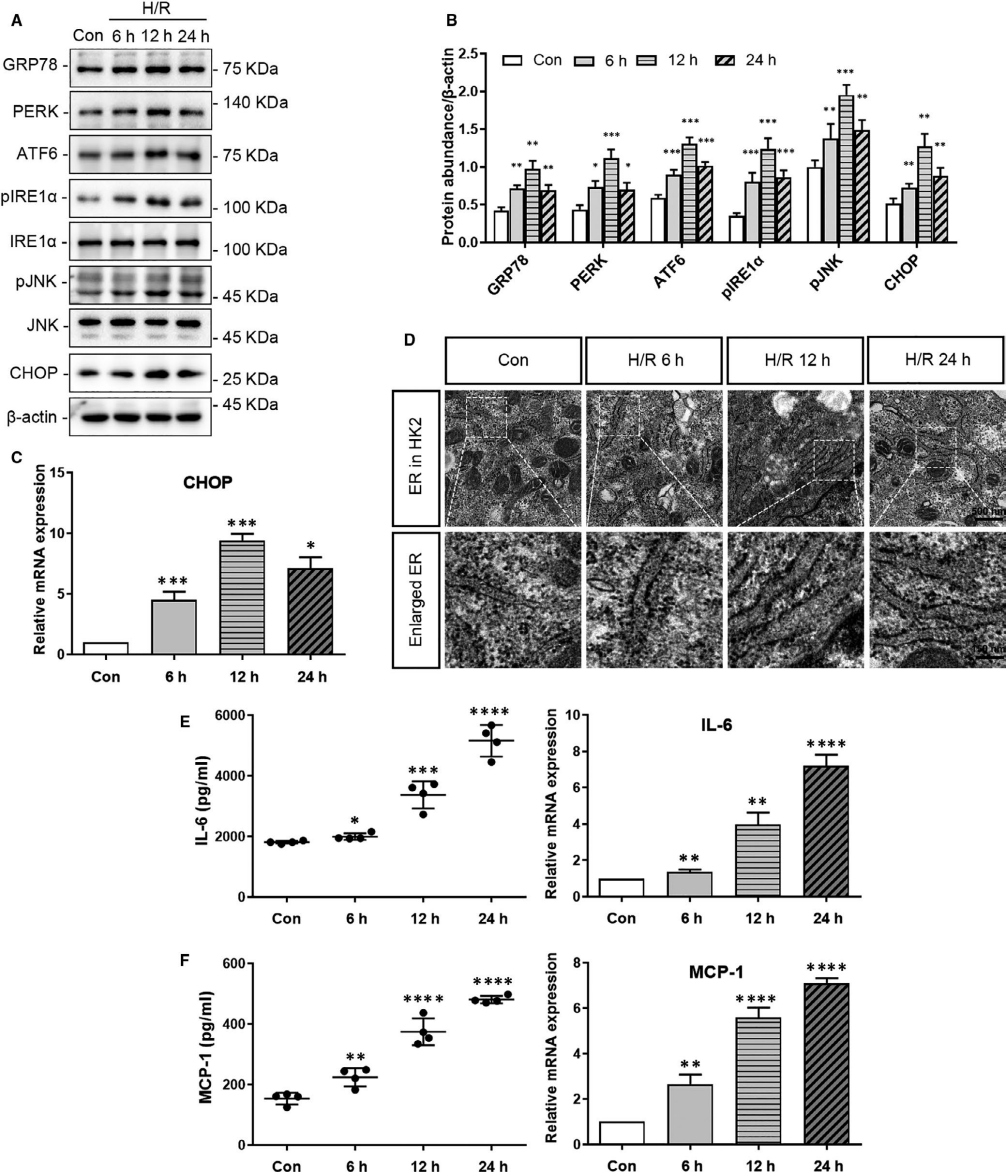
Hypoxia‐reoxygenation (H/R) induced ER stress and the production of inflammatory cytokines in HK‐2 cells. HK‐2 cells were subjected to H/R (4 h of hypoxia followed by 6, 12 or 24 h reoxygenation). Cells were collected for western blot analysis, qRT‐PCR or observation by transmission electron microscopy (TEM) analyses, and culture supernatants were collected for ELISA. The controls were cultures without H/R. A, Representative western blots of ER stress markers (GRP78, PERK, ATF6, pIRE1α, pJNK, CHOP), and β‐actin (loading control). B, Quantitative analysis of western blots of ER stress markers (GRP78, PERK, ATF6, pIRE1α, pJNK, CHOP). C, qRT‐PCR analysis of *CHOP* and *ACTB* (internal control). D, Observation of the ER via TEM. E, IL‐6 secretion to the culture supernatant, and qRT‐PCR analysis of *IL‐6* and *ACTB* (internal control). F, MCP‐1 secretion to the culture supernatant, and qRT‐PCR analysis of *MCP‐1* and *ACTB* (internal control). Con: the control group; 6 h: 4 h of hypoxia followed by 6 h reoxygenation; 12 h: 4 h of hypoxia followed by 12 h reoxygenation; 24 h: 4 h of hypoxia followed by 24 h reoxygenation. (*P < .05, **P < .01, ***P < .001 vs Control Group)

Endoplasmic reticulum stress was investigated in HK‐2 cells exposed to H/R. As shown in Figure 1A,B, the expression of ER stress markers (GRP78, PERK, ATF6, pIRE1α, pJNK and CHOP) increased significantly at 6, 12 and 24 hours of reoxygenation following 4 hours hypoxia, compared with that in the control (at least *P* < .05). Moreover, qRT‐PCR analysis revealed a significantly increased expression of *CHOP* mRNA in HK‐2 cells at 6, 12 and 24 hours of reoxygenation following 4 hours of hypoxia (at least *P* < .05) compared with that in control (Figure 1C). Importantly, transmission electron microscopy showed that the width of ER in HK‐2 cells increased after H/R (Figure 1D).

Inflammatory cytokines in HK‐2 cells also increased after H/R. As shown in Figure 1E,F IL‐6 and MCP‐1 protein and mRNA expression increased significantly in H/R HK‐2 cells, compared with that in the control (at least *P* < .05). Of note, we observed that enhanced expression of IL‐6 and MCP‐1 in HK‐2 cells was directly proportional to reoxygenation time following 4 hours of hypoxia (Figure 1E,F).

### 3.2 The IRE1α/JNK1/CHOP pathway regulates ER stress and inflammatory cytokine production in HK‐2 cells following H/R

**FIGURE 2:**
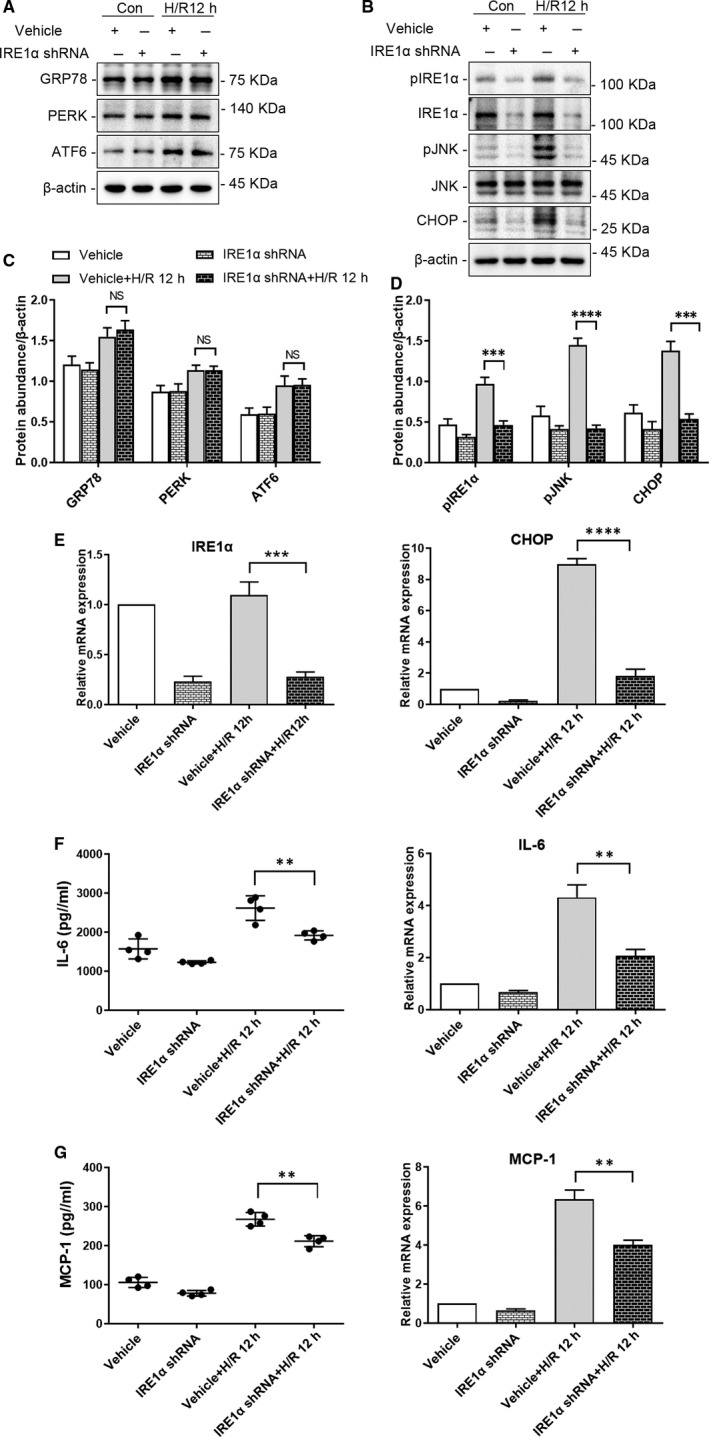
Depletion of IRE1α reduces ER stress and the production of inflammatory cytokines in HK‐2 cells following hypoxia‐reoxygenation (H/R). HK‐2 cells depleted of IRE1α and vehicle‐infected HK‐2 cells were cultured under normal conditions or subjected to H/R (4 h of hypoxia followed by 12 h reoxygenation). Representative western blots of ER stress markers (A) upstream (GRP78, PERK, ATF6) and (B) downstream (pJNK, CHOP) of IRE1α; β‐actin was used as the loading control. Quantitative analysis of western blots of ER stress markers (C) upstream (GRP78, PERK, ATF6) and (D) downstream (p‐JNK, CHOP) of IRE1α. E, qRT‐PCR analysis of *IRE1α*, *CHOP* and *ACTB* (internal control). F, IL‐6 secretion to culture supernatant, and qRT‐PCR analysis of *IL‐6* and *ACTB* (internal control). G, MCP‐1 secretion to the culture supernatant, and qRT‐PCR analysis of *MCP‐1* and *ACTB* (internal control). Con: HK‐2 cells without H/R; H/R 12 h:HK‐2 cells with 4 h of hypoxia followed by 12 h reoxygenation; IRE1αShRNA: IRE1α shRNA‐infected HK‐2 cells; Vehicle: vehicle‐infected HK‐2 cells. (NS: not significant, **P < .01, ***P < .001, ****P < .0001)

We knocked down (shRNA‐mediated) different signalling molecules to elucidate their role in the responses to H/R in HK‐2 cells. Lentiviral transduction with an IRE1α‐targeting shRNA significantly down‐regulated the expression of IRE1α as well as that of downstream molecules(pJNK and CHOP) in the ER stress pathway of HK‐2 cells (Figure 2B,D, pJNK: *P* < .0001; CHOP: *P* < .001). qRT‐PCR analysis also revealed a significant decrease in the *CHOP* mRNA levels in IRE1α shRNA‐treated HK‐2 cells following 12 hours of H/R (Figure 2E, *P* < .0001) compared with that in vehicle‐infected cells. In contrast, no significant change was observed in the expression of molecules upstream of IRE1α in the ER stress pathway (GRP78, PERK, ATF6) in HK‐2 cells infected with IRE1α shRNA vs vehicle(Figure 2A,C, *P* > .05). Furthermore, knock‐down of IRE1α in HK‐2 cells significantly down‐regulated protein synthesis and mRNA expression of the inflammatory cytokines IL‐6 and MCP‐1 (Figure 2F,g, *P* < .01), compared to those in vehicle‐infected HK‐2 cells, following 12 hours of H/R.

**FIGURE 3:**
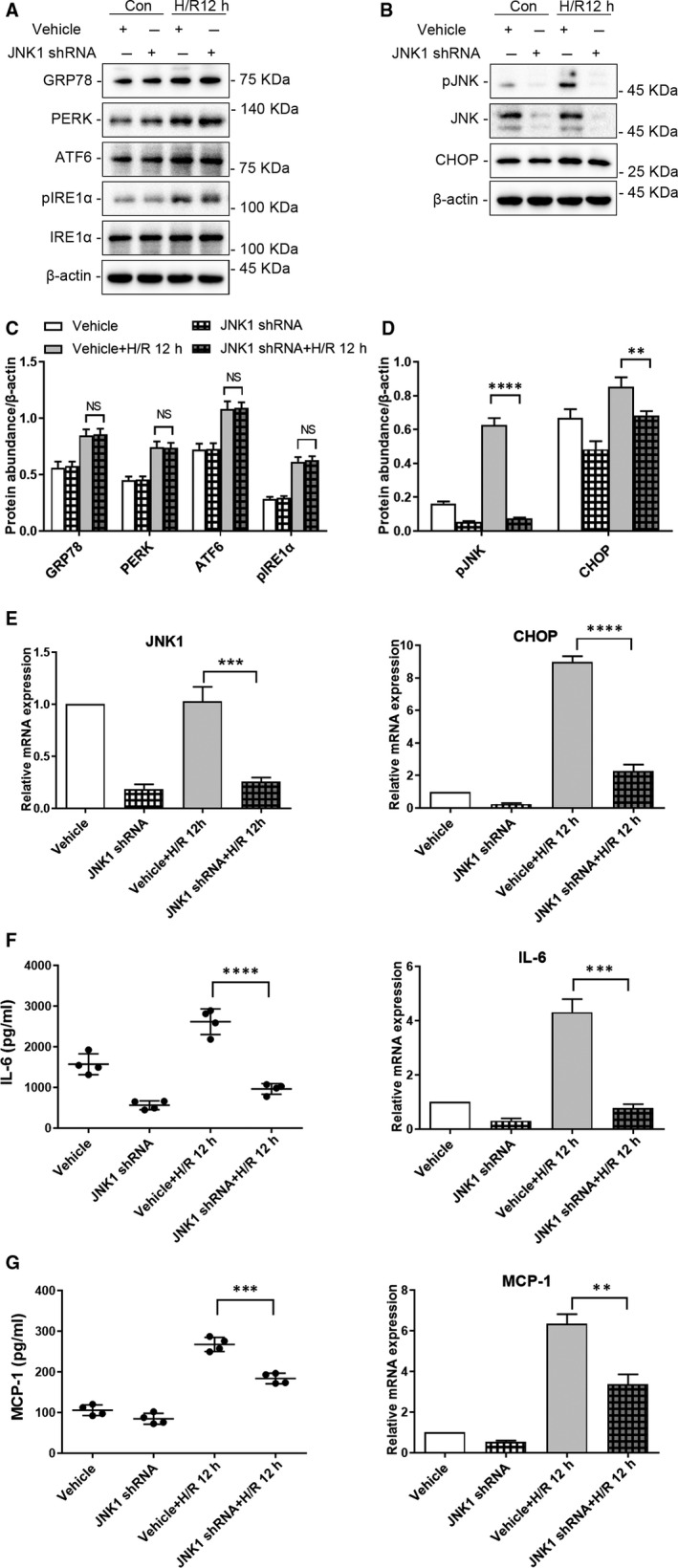
Depletion of JNK1 reduces ER stress and the production of inflammatory cytokines in HK‐2 cells following hypoxia‐reoxygenation (H/R). HK‐2 cells depleted of JNK1 and vehicle‐infected HK‐2 cells were cultured under normal conditions or subjected to H/R (4 h of hypoxia followed by 12 h reoxygenation). Representative western blots of ER stress markers (A) upstream (GRP78, PERK, ATF6, pIRE1α) and (B) downstream (CHOP) of JNK; β‐actin was used as the loading control. Quantitative analysis of western blots of ER stress markers (C) upstream (GRP78, PERK, ATF6, pIRE1α) and (D) downstream (CHOP) of JNK. E, qRT‐PCR analysis of *JNK1*, *CHOP* and *ACTB* (internal control). F, IL‐6 secretion to the culture supernatant, and qRT‐PCR analysis of *IL‐6* and *ACTB* (internal control). G, MCP‐1 secretion to the culture supernatant, and qRT‐PCR analysis of *MCP‐1* and *ACTB* (internal control). Con: HK‐2 cells without H/R; H/R 12 h: HK‐2 cells with 4 h of hypoxia followed by 12 h of reoxygenation; JNK1 ShRNA: JNK1 shRNA‐infected HK‐2 cells; Vehicle: vehicle‐infected HK‐2 cells.(NS: not significant, **P < .01,***P < .001, ****P < .0001)

Similarly, compared with vehicle‐infected HK‐2 cells following 12 hours of H/R, lentiviral transduction with an shRNA targeting JNK1 significantly down‐regulated JNK and CHOP in HK‐2 cells (Figure 3B,D, pJNK: *P* < .0001; CHOP: *P* < .01). qRT‐PCR analysis also revealed a significant decrease of *CHOP* mRNA levels in JNK1 shRNA‐treated HK‐2 cells following 12 hours of H/R (Figure 3E, *P* < .0001) compared to that in vehicle‐infected cells. Of note no significant change was observed in the expression of molecules upstream of JNK in the ER stress pathway (GRP78, PERK, ATF6, pIRE1α) (Figure 3A,C, *P* > .05). Furthermore, knock‐down of JNK1 in HK‐2 cells significantly down‐regulated inflammatory cytokines, IL‐6 and MCP‐1 protein and mRNA (Protein: IL‐6: *P* < .0001, MCP‐1: *P* < .001; mRNA: IL‐6: *P* < .001, MCP‐1: *P* < .01) compared to those in vehicle‐infected HK‐2 cells (Figure 3F,G).

### 3.3 IL‐6 induced activation of STAT3 and ECM production in HRMC

**FIGURE 4:**
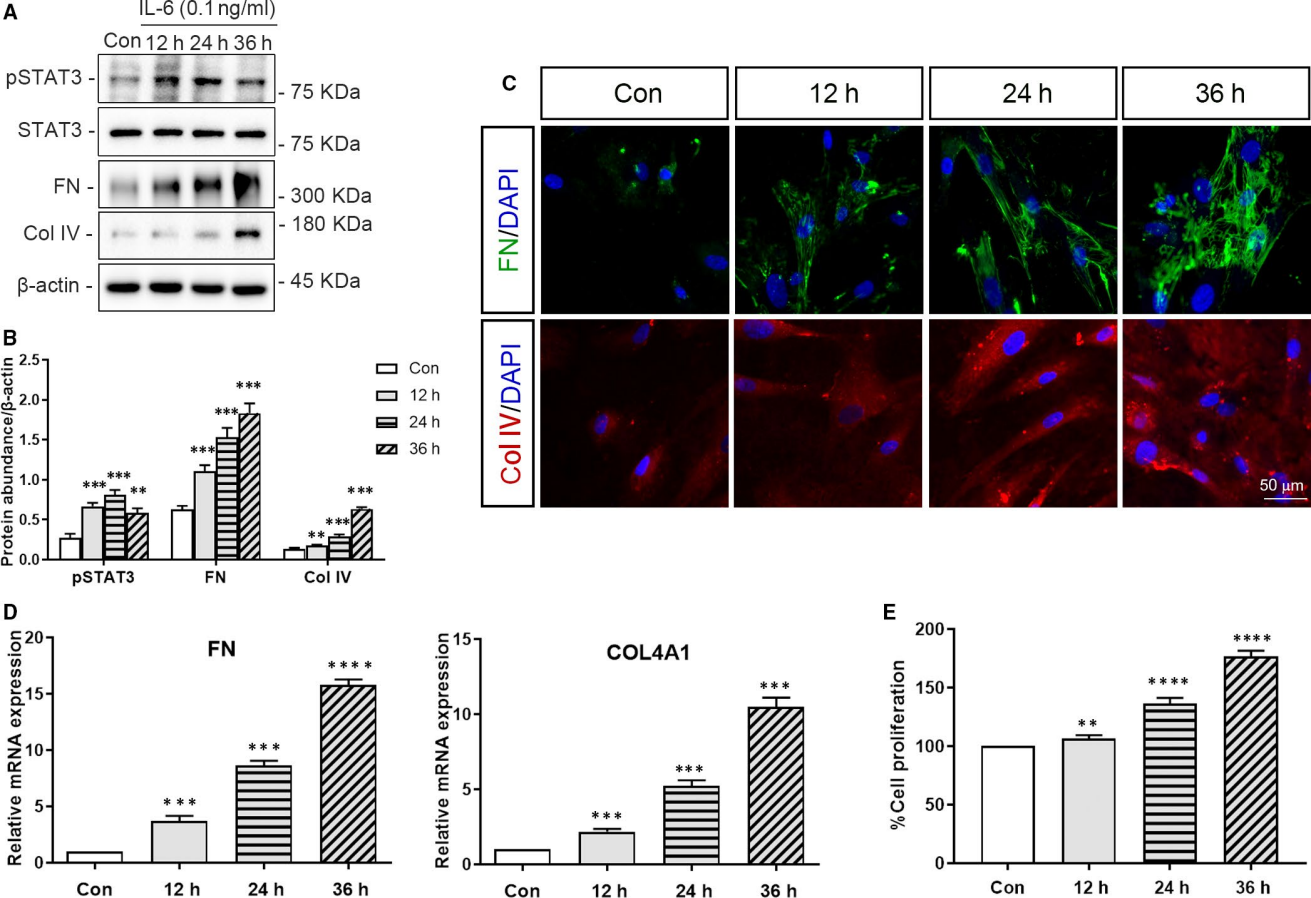
Expression of pSTAT3 and ECM in HRMC and the proliferation of IL‐6 stimulated HRMC. HRMC were incubated in media with or without IL‐6 (0.1 ng/mL) for 12, 24 or 36 h. The levels of pSTAT3 and the main components of ECM (FN and Col IV) in HRMC were analysed via western blot analysis, immunofluorescence or qRT‐PCR. The proliferation of HRMC was assessed using the cell counting kit‐8 (CCK‐8) assay using a microplate reader. A, Representative western blots of pSTAT3, FN, Col IV, and β‐actin (loading control). B, Quantitative analysis of western blots of pSTAT3, FN, and Col IV. C, Representative immunofluorescence images of FN, COL IV and DAPI. D, qRT‐PCR analysis of *FN*,*COL4A1* and *ACTB* (internal control). E, CCK‐8 analysis of the proliferation of HRMC. Con: the control group, 12 h: HRMC stimulated with IL‐6 for 12 h; 24 h: HRMC stimulated with IL‐6 for 24 h; 36 h: HRMC stimulated with IL‐6 for 36 h. (**P < .01,***P < .001, ****P < .0001 vs Control Group)

Human renal mesangial cell treated with IL‐6 for 12, 24 and 36 hours showed significant increase in expression of p‐STAT3, and the main components of ECM (FN and Col IV), compared with those in control cells(at least *P* < .01 vs control) (Figure 4A,B). The immunofluorescence analysis of FN and Col IV showed the same results (Figure 4C). qRT‐PCR analysis also revealed an increase in *FN* and *COL4A1* mRNA expression in HRMC following the stimulation with IL‐6 (FN at 36 hours: *P* < .0001, others: *P* < .001 vs control) (Figure 4D).

IL‐6 also induced a significant, and time‐dependent increase in the proliferation of HRMC compared with that of control cells (12 hours: *P* < .01, others: *P* < .0001 vs control) (Figure 4E).

**FIGURE 5:**
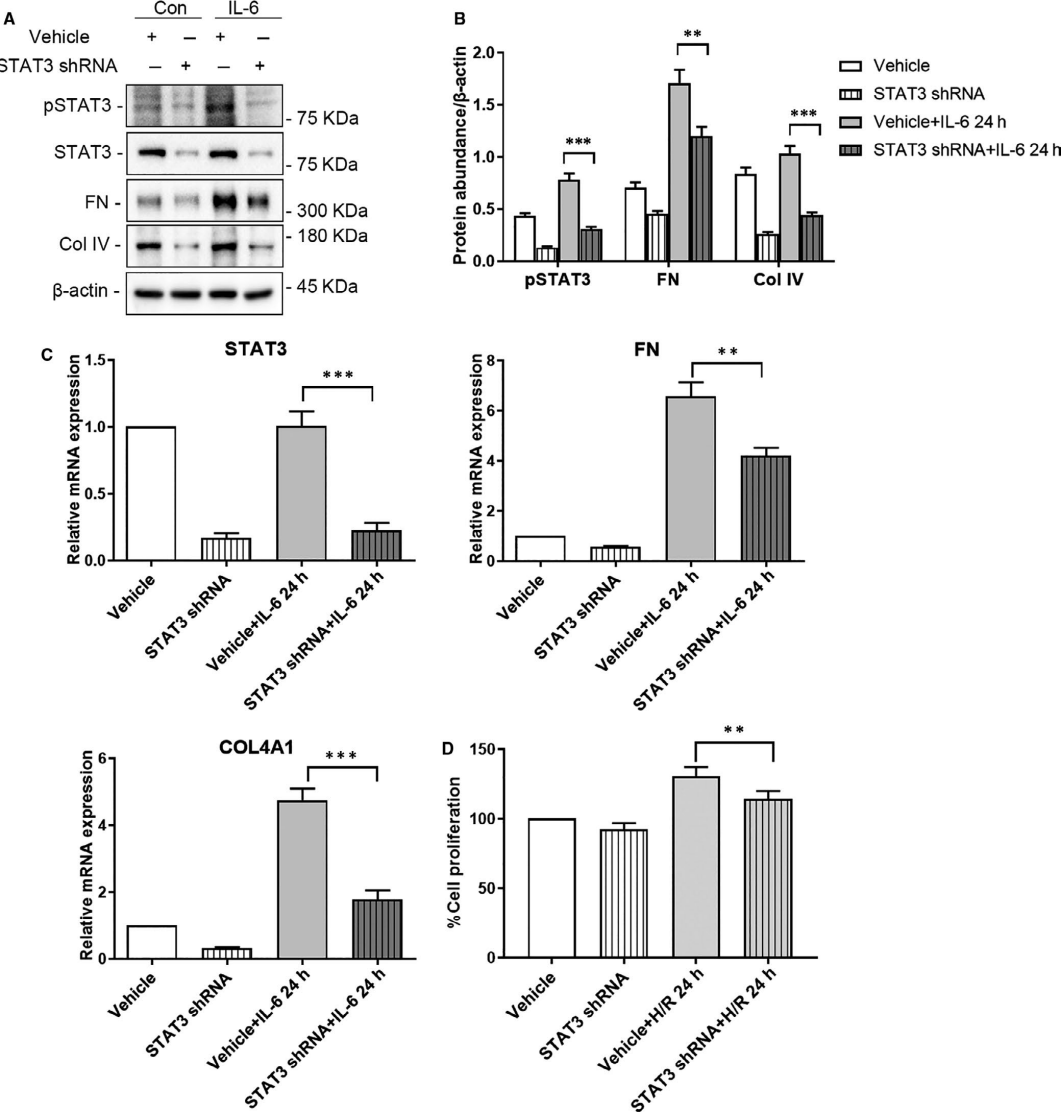
Depletion of STAT3 reduced the release of ECM and the proliferation of HRMC stimulated with IL‐6. HRMC transfected with STAT3 shRNA adenovirus or vehicle adenovirus were incubated in medium with or without IL‐6 (0.1 ng/mL) for 24 h. A, Representative western blots of pSTAT3, FN, Col IV, and β‐actin (loading control). B, Quantitative analysis of western blots of pSTAT3, FN, and Col IV. C, qRT‐PCR analysis of *STAT3*, *FN*, *COL4A1* and *ACTB* (internal control). D, CCK‐8 analysis of the proliferation of HRMC. Con: HRMC without IL‐6 stimulation; IL‐6: HRMC with IL‐6 stimulation for 24 h; STAT3 ShRNA: STAT3 shRNA‐treated HRMC; Vehicle: vehicle‐treated HRMC. (**P < .01, ***P < .001)

To confirm that the effects of IL‐6 in HRMC were mediated by STAT3 activation, we knocked down the expression of STAT3 in HRMC via adenovirus‐mediated shRNA transduction (Figure 5A–D). Depletion of STAT3 significantly inhibited the ECM components FN and Col IV protein and mRNA in IL‐6‐treated HRMC compared to that in IL‐6‐treated, vehicle‐transfected HRMC (Figure 5A–C) (Protein: FN: *P* < .01, others: *P* < .001; mRNA: FN: *P* < .01, others: *P* < .001). Similarly, the IL‐6 induced proliferation was also significantly lower in STAT3‐depleted HRMC vs vehicle‐transfected HRMC (*P* < .01; Figure 5D).

### 3.4 Depletion of IRE1/JNK in HK‐2 cells attenuates HRMC proliferation in the context of co‐cultures, in the response to 24 hour H/R

**FIGURE 6:**
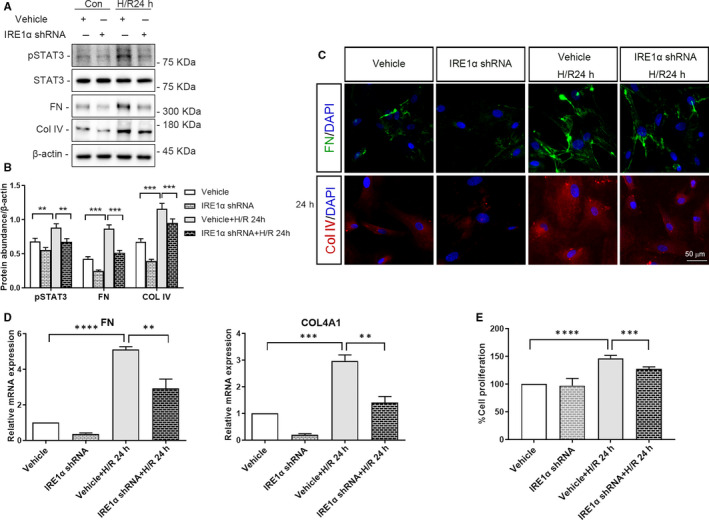
Depletion of IRE1α in HK‐2 cells reduces the expression of pSTAT3, the release of ECM and the proliferation of co‐cultured HRMC. HK‐2 cells depleted of IRE1α or vehicle‐infected HK‐2 cells subjected to or not to hypoxia for 4 h were co‐cultured with HRMC for 24 h in trans‐well pates under normal culture conditions. A, Representative western blots of pSTAT3, FN, Col IV, and β‐actin (loading control). B, Quantitative analysis of western blots of pSTAT3, FN, and Col IV. C, Representative immunofluorescence images of FN, COL IV and DAPI. D, qRT‐PCR analysis of *FN*, *COL4A1* and *ACTB* (internal control). E, CCK‐8 analysis of the proliferation of HRMC. Con: HK‐2 cells without H/R; H/R 24 h: HK‐2 cells with 4 h of hypoxia followed by 24 h reoxygenation; IRE1α ShRNA: IRE1α shRNA‐infected HK‐2 cells; Vehicle: vehicle‐infected HK‐2 cells. (**P < .01, ***P < .001, ****P < .0001)

**FIGURE 7:**
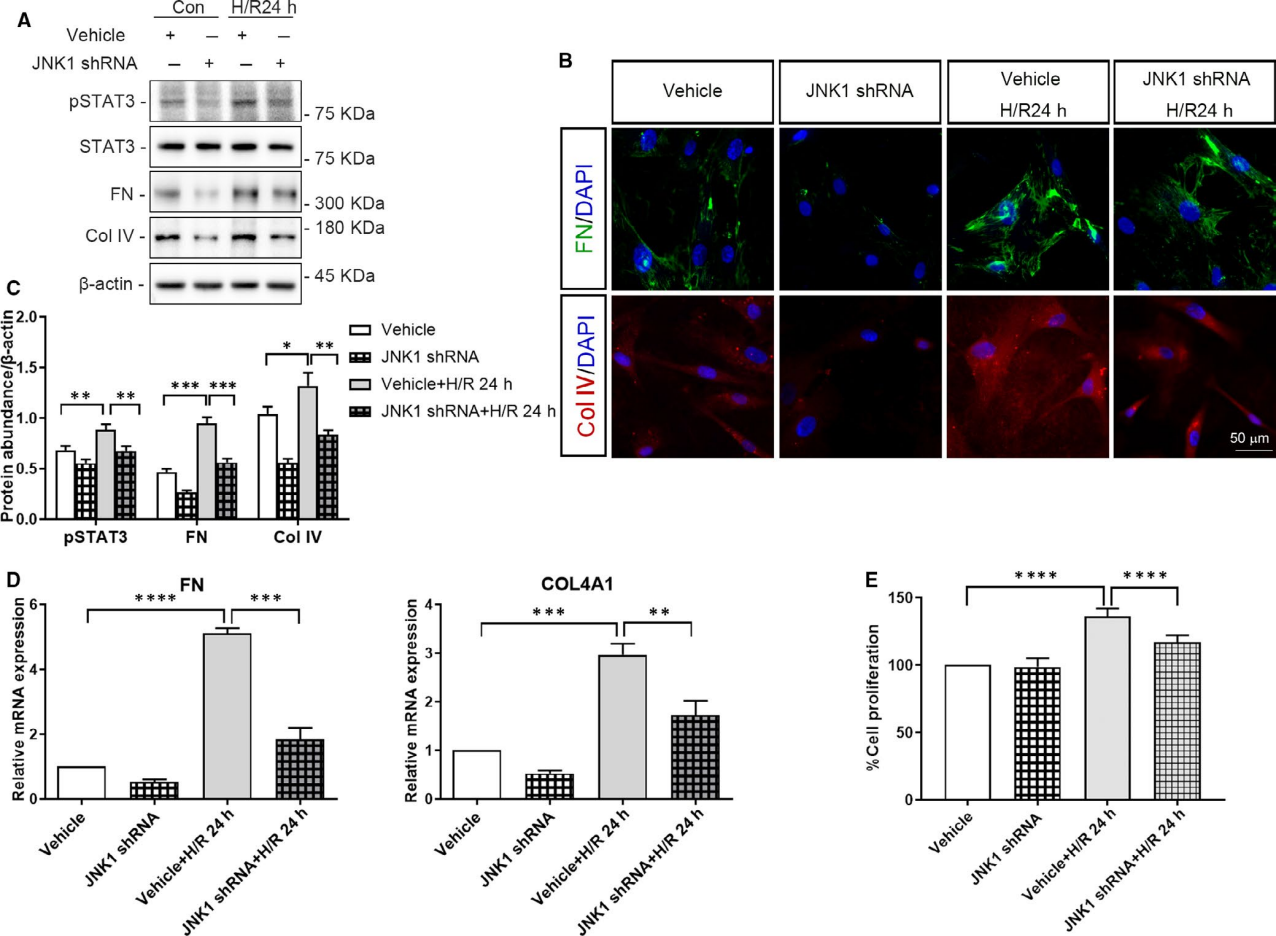
Depletion of JNK1 in HK‐2 cells reduces the expression of pSTAT3, the release of ECM and the proliferation of co‐cultured HRMC. HK‐2 cells depleted of JNK1 or vehicle‐infected HK‐2 cells subjected to or not to hypoxia for 4 h co‐cultured with HRMC for 24 h in trans‐well pates under normal culture conditions. A, Representative western blots of pSTAT3, FN, Col IV, and β‐actin (loading control). B, Quantitative analysis of western blots of pSTAT3, FN, and Col IV. C, Representative immunofluorescence images of FN, COL IV and DAPI. D, qRT‐PCR analysis of *FN*, *COL4A1* and *ACTB* (internal control). E, CCK‐8 analysis of the proliferation of HRMC. Con: HK‐2 cells without H/R; H/R 24 h:HK‐2 cells with 4 h of hypoxia followed by 24 h reoxygenation; JNK1 ShRNA: JNK1 shRNA‐treated HK‐2 cells; Vehicle: vehicle‐treated HK‐2 cells. (**P < .01, ***P < .001, ****P < .0001)

Proliferation of HRMC was significantly higher in the context of co‐culture with vehicle‐infected HK‐2 cells following 24 hours H/R, than that in the context of no H/R (Figure 6D and 7D, *P* < .0001). Moreover, shRNA‐mediated depletion of IRE1α or JNK1 in HK‐2 cells, via lentiviral transduction, attenuated the proliferation of co‐cultured HRMC following 24 hours of H/R (Figure 6D and 7D, IRE1α‐depletion: *P* < .001, JNK1‐depletion: *P* < .0001).

### 3.5 Depletion of IRE1/JNK in HK‐2 cells decrease STAT3 phosphorylation and ECM deposition in co‐cultured HRMC, following 24 hour of H/R

The protein expression of pSTAT3, FN and Col IV in HRMC co‐cultured with vehicle‐infected HK‐2 cells following 24 hours of H/R was significantly higher than that in HRMC co‐cultured with vehicle‐infected HK‐2 cells not subjected to H/R (at least *P* < .05). Moreover, FN and Col IV immunofluorescence revealed the same results (Figures 6B and 7B). *COL4A1 and FN* mRNA levels were also significantly increased in HRMC co‐cultured with vehicle‐infected HK‐2 cells in the context of 24 hours of H/R vs no H/R (Figure 6C and 7C, *FN*: *P* < .0001, *COL4A1*: *P* < .001).

In addition, protein and mRNA levels of FN and Col IV in HRMC were significantly decreased upon co‐culture with IRE1α‐ or JNK1‐depleted HK‐2 cells following 24 hours H/R, compared to those in HRMC co‐culture with vehicle‐infected HK‐2 cells following 24 hours of H/R (Figure 6A–C and 7A–C, at least *P* < .01).

## 4 DISCUSSION

Acute kidney injury is a pathological condition characterized by the injury and subsequent death of renal tubular epithelial cells.^14^, ^24^, ^25^ TEC, a specialized tubular segment adjacent to the glomerulus, is not only the target of injury but also an important mediator of renal fibrosis progression.^26^ Several responses to kidney injury are prone to trigger ER stress in AKI.^12^ ER stress also contributes critically to the development of chronic kidney pathologies and CKD following AKI. Importantly, inhibition of ER stress may represent a potential therapeutic strategy to impede AKI‐CKD transition.^2^ In ER stress, the IRE1/ JNK pathway is the most closely related to inflammation, IRE1 induces the activation of JNK, which in turn promotes inflammatory responses.^1^ In this study, we used H/R HK‐2 cells as a model of AKI and found that markers of ER stress (GRP78, PERK, ATF6, pIRE1, pJNK, CHOP) were significantly increased in H/R HK‐2 cells vs control cells.

A growing number of epidemiologists have suggested that the incomplete recovery from AKI leads to progressive CKD.^27^, ^28^, ^29^, ^30^ The possible behind mechanisms include ER stress, incomplete or maladaptive tissue repair, driven fibrosis, inflammation and capillary rarefaction.^8^, ^31^, ^32^, ^33^ The loss of nephron mass and the consequent nephron hyperfiltration can lead to the activation of the renin‐angiotensin system, hypertension and the subsequent glomerulosclerosis.^34^, ^35^, ^36^ Experimental models have shown that selective epithelial injury could drive capillary sparseness, interstitial fibrosis, glomerulosclerosis and CKD, substantiating a direct role for damaged TEC in disease progression.^37^ Therefore, recent research on AKI/CKD has focused on the functions of TEC.^6^, ^37^ Now, it is clearly evident that ER stress and inflammatory response actively targets renal cells during the development of AKI and progression to CKD.^3^, ^12^ Furuichi et al reported that MCP‐1/CCL2 was one of the most widely studied chemokines in AKI and CKD.^38^ In this study, the IRE1/JNK pathway induced the secretion of inflammatory cytokines such as IL‐6 and MCP‐1 in H/R HK‐2 cells. Moreover, shRNA‐mediated depletion of IRE1α or JNK1 significantly reduced ER stress and the production of IL‐6 and MCP‐1 in H/R HK‐2 cells. These results implicated the IRE1/JNK pathway in the activation of ER stress and inflammatory responses observed in AKI.

Mesangial cell proliferation and excessive deposition of ECM proteins are important aspects of glomerulosclerosis. Mesangial cells are an essential component of the glomerulus and have several biological functions. Since they are the main producers of ECM in renal tissues, they are also behind the promotion of glomerulosclerosis.^39^ It has been shown that inflammatory factors could damage mesangial cells, promote their proliferation and increase ECM in several ways. For instance, MCP‐1 can bind to its receptor, CCR2, on mesangial cells, activate p38 mitogen activated protein kinase signalling, promote cell proliferation and regulate endothelin‐1 to increase the secretion of ECM.^23^, ^40^ Recently, it was shown that the activation of the IL‐6/STAT3 pathway in the renal interstitium and various tissues could promote fibrosis and sclerosis.^20^, ^21^ Therefore, the IL‐6/STAT3 pathway may mediate mesangial cells inflammatory damage and induce the increased deposition of glomerular matrix proteins in AKI.

Tubulointerstitial damage may act as a sensitize to subsequent glomerular damage. In fact, in a diabetic nephropathy in vitro model, tubulointerstitial injury appeared to affect the progression of glomerular lesions.^41^ Grgic et al reported that epithelial cells treated with three doses of diphtheria toxin led to persistent inflammation, microvascular rarefaction, increased collagen α‐1(I), TGF‐β1 and FN levels, progressive fibrogenesis and secondary glomerulosclerosis.^8^ Therefore, it was suggested that damaged renal tubular epithelial cells could induce glomerulosclerosis, however, the underlying mechanism remained unknown. Our study showed that following H/R, ER stress was associated with the reduced expression of inflammatory cytokines in IRE1α‐ or JNK1‐depleted cells compared to that in the vehicle‐infected HK‐2 cells. Notably, we found that IL‐6 activated the STAT3 pathway and indirectly promoted the secretion of ECM by HRMC in the context of co‐cultures with H/R HK‐2 cells. Moreover, in the same co‐culture system, the inhibition of the IRE1/JNK pathway in H/R HK‐2 cells attenuated STAT3 signalling and ECM production in HRMC. Overall, these data suggested that the inhibition of the IRE1/JNK pathway may attenuate glomerulosclerosis in damaged TEC.

In addition, there are also some studies focus on influence from glomerular cells on TEC. Chan et al^42^ found that inflammatory cytokines released from mesangial cells could alter the glomerular barrier pore size, allowing the passage of inflammatory mediators to the TEC in IgA nephropathy. Moreover, Jeon et al^43^ suggested that miRNAs in extracellular vesicles from injured podocytes could lead to tubular epithelial cells’ damage.

Our study is not without limitations. We used a simple AKI cell‐based in vitro model and tested the direct effects of TEC on mesangial cells, whereas the mechanisms of AKI‐CKD transition are very complex. Further studies are needed to validate the mechanism of TEC‐induced glomerulosclerosis in vivo, using the relevant animal models and then clinically (in human patients). It is also necessary to identify the molecules involved in the TEC‐mediated regulation of mesangial cell functions in AKI‐CKD transition.

In conclusion, we demonstrated that the IRE1/JNK pathway was activated in H/R HK‐2 cells and induced the secretion of inflammatory cytokines such as IL‐6 and MCP‐1. Moreover, the activation of this pathway in H/R HK‐2 cells contributed to the maladaptation of mesangial cells, leading to STAT3‐mediated cell proliferation and ECM secretion. IRE1α or JNK1 knock‐down in H/R HK‐2 cells attenuated the CKD‐related phenotype in mesangial cells, regulating the production of ECM. Together, these findings suggest that targeting IRE1 or JNK may be a useful strategy to treat for glomerulosclerosis and to prevent AKI‐CKD transition.

## CONFLICT OF INTEREST

The authors confirm that there are no conflicts of interest to declare.

## AUTHOR CONTRIBUTION

**Yan Liang:** Conceptualization (equal); Funding acquisition (lead); Project administration (equal); Writing‐original draft (equal); Writing‐review & editing (equal). **Lulu Liang:** Methodology (lead); Writing‐original draft (equal); Writing‐review & editing (equal). **Zhenjie Liu:** Methodology (supporting). **Yingzi Wang:** Methodology (supporting). **Xiubing Dong:** Methodology (supporting). **Lingyun Qu:** Methodology (supporting). **Rong Gou:** Methodology (supporting). **Yulin Wang:** Methodology (supporting). **Qian Wang:** Methodology (supporting). **Zhangsuo Liu:** Conceptualization (equal); Funding acquisition (equal); Project administration (equal); Supervision (equal). **Lin Tang:** Conceptualization (equal); Project administration (equal); Supervision (equal).

## Data Availability

The data in this study are openly available in a public repository.
